# Using applied social science disciplines to implement creative outdoor cat management solutions and avoid the trap of one‐size‐fits‐all policies

**DOI:** 10.1111/cobi.14321

**Published:** 2024-07-08

**Authors:** Kirsten Mya Leong, Ashley Rochelle Gramza, Jennifer N. Duberstein, Chelsey Bryson, Angela Amlin

**Affiliations:** ^1^ NOAA Pacific Islands Fisheries Science Center Honolulu Hawaii USA; ^2^ Playa Lakes Joint Venture Erie Colorado USA; ^3^ Sonoran Joint Venture Tucson Arizona USA; ^4^ Hawaiian Humane Society Honolulu Hawaii USA; ^5^ NOAA Pacific Islands Regional Office Honolulu Hawaii USA

**Keywords:** domestic cats, management dimensions, mental models, panacea traps, value‐based conflicts, conflictos basados en valores, dimensiones del manejo, gatos domésticos, modelos mentales, trampas panacea

## Abstract

In the United States, policy conflicts have prevented successful population‐level management of outdoor cats for decades. Wildlife conservation professionals have sought widespread use of humane dispatch (i.e., lethal culling applied humanely), whereas cat welfare professionals have promoted trap–neuter–return (TNR) (cats are trapped, neutered, and returned to the outdoors). These conflicts represent a policy panacea trap, which we argue drives many conservation conflicts. In these situations, the focus on defending a one‐size‐fits‐all policy fails to account for the value differences that shape the different understandings of the problem and desired outcomes associated with each policy, as well as complexities in the social–ecological system. Over the past 5 years, a group of wildlife conservation and cat welfare professionals codeveloped a set of products that have started to be used to help organizations break out of the policy panacea trap. We used a case study to illustrate how efforts grounded in applied social science disciplines, such as science communication, social–ecological systems, and conservation marketing, can help identify a more robust set of policy options tailored to local management and cultural contexts for successful implementation. Shifting the focus to embrace a shared understanding of the broader system helped us identify areas for collaboration, broaden the policy toolbox, and allow space for policy tools originally framed as opposing panaceas. This work helped prepare all parties to have difficult but productive discussions and address shared policy needs. We suggest that many value‐based conservation conflicts would benefit from similar efforts that use applied social science to transform how conflict is addressed, moving beyond policy panaceas that end in stalemate to develop shared understandings of context‐specific policies, and to identify opportunities for creative cooperation that yield real conservation progress.

## Abstract (es)

Uso de las disciplinas aplicadas de las ciencias sociales para implementar soluciones creativas de manejo de gatos callejeros y evitar la trampa de las políticas universales

**Resumen:**

Durante décadas, los conflictos entre políticas han evitado un manejo exitoso a nivel poblacional de los gatos callejeros en los Estados Unidos. Los profesionales de la conservación silvestre han buscado el uso extenso de los sacrificios con humanidad, mientras que los profesionales del bienestar felino han promovido la captura‐esterilización‐liberación (CEL) de los gatos. Estos conflictos representan una trampa panacea de políticas, la cual argumentamos causa muchos conflictos de conservación. En estas situaciones, el enfoque en defender una política universal no logra considerar la diferencia de valores que forman los diferentes entendimientos del problema y los resultados deseados que se asocian con cada política, así como las complejidades dentro del sistema socio‐ecológico. A lo largo de los últimos cinco años, un grupo de profesionales de la conservación silvestre y del bienestar felino desarrollaron en conjunto una serie de productos que han comenzado a ayudar a las organizaciones a salir de la trampa panacea de políticas. Usamos un estudio de caso para ilustrar cómo los esfuerzos cimentados en las disciplinas aplicadas de las ciencias sociales (p. ej.: las ciencias de la comunicación, los sistemas socio‐ecológicos y el marketing de la conservación) pueden ayudar a identificar un conjunto más sólido de opciones de políticas personalizadas para el manejo local y los contextos culturales para tener una implementación exitosa. El cambio de enfoque para aceptar el entendimiento compartido del sistema más amplio nos ayudó a identificar áreas de colaboración, a ampliar las herramientas para las políticas y a permitirle espacio a las herramientas formuladas originalmente como panaceas contrarias. Este trabajo ayudó a que todas las partes se prepararan para tener discusiones difíciles pero productivas y para abordar las necesidades compartidas de las políticas. Sugerimos que muchos conflictos de conservación basados en los valores se beneficiarían de un esfuerzo similar que use las ciencias sociales aplicadas para transformar cómo se aborda el conflicto, llegando más allá de las panaceas de políticas que terminan en un punto muerto para el desarrollo del entendimiento compartido de políticas específicas al contexto, y para identificar las oportunidades de cooperación creativa que producen un progreso real de la conservación.

## OUTDOOR CAT CONFLICTS AND POLICY PANACEA TRAPS

In January 2022, 2 bills were introduced in the Hawaii state legislature that represent the intractable, value‐laden policy conflicts that have prevented effective population management of free‐roaming outdoor domestic cats (*Felis catus*) in the United States for decades. One would require the state agency to count and cull feral cats and consider using poison baits as a culling technique (HB1987). Wildlife conservation professionals have argued for lethal methods of culling applied humanely (hereafter referred to as humane dispatch) to reduce cat populations, calling into question the logic of those who favor other methods as “merchants of doubt” (Loss & Marra, 2018). The other bill (SB2837) would fund low‐cost spaying or neutering as a means of population control, with a large part of the funds used to sterilize free‐roaming cats and return them outdoors, a technique known as trap–neuter–return (TNR). Cat welfare professionals argue that the claims against TNR are overblown and represent a “moral panic over cats” (Lynn et al., 2019). These ongoing conflicts have been described as “cat wars” (Marra & Santella, 2016), with each side publishing numerous studies supporting their preferred policy solution and exposing the perceived flaws in the other side's reasoning (reviewed by Wald & Peterson [2020]). These conflicts represent a policy panacea trap, where the focus on defending a one‐size‐fits‐all single policy to address a wide range of issues exacerbates conflicts and prevents parties in conflict from finding ways to move forward together to devise solutions that may require a mix of policies.

Elinor Ostrom and colleagues examined the tendency of scholars and practitioners to rely on policy panaceas that can impede effective governance of complex and multifaceted problems in social–ecological systems (SESs) (Ostrom, 2007; Ostrom et al., 2007). Although Ostrom's work focuses on common pool resources, we have observed that many other types of conservation conflict also have this tendency to advocate a one policy solution and deride “alternatives…as worse than useless” (Brock & Carpenter, 2007, p. 15206). These conflicts are often social conflicts at their core, based on divergent value systems. Some examples include conservation conflicts over deforestation and agriculture, predator recovery, and the efficacy of no‐take policies for management of marine protected areas (Cumming, 2018; Levin et al., 2021; Nie, 2003).

Panacea traps arise from a false assumption that most people's preferences are the same and therefore a single policy solution can represent the same optimal truth for all people and all situations (Brock & Carpenter, 2007; Ostrom et al., 2007). Breaking free from this assumption is especially important for value‐driven problems that include multiple groups of people who view the problem differently. Research on wicked problems shows that in these situations “…formulation of a wicked problem *is* the problem” (Rittel & Webber, 1973, p. 161) because the people in conflict use different moral and philosophical standards to assess the problem and appropriateness of proposed solutions (Balint et al., 2011; Leong et al., 2020; Rittel & Webber, 1973). Further, addressing one piece of the problem can inadvertently activate or intensify other facets of the problem and new groups of people. Rather than an optimal policy panacea that works across all contexts, managing these problems requires understanding the range of potential policy solutions, identifying the mix most appropriate to the specific social–ecological context, and adapting strategies through time as problems evolve and change (Balint et al., 2011; Cumming, 2018).

Conservation science is a value‐laden, mission‐oriented discipline (Mascia et al., 2003; Noss, 2007), so it is unsurprising that many conservation conflicts, especially human–wildlife conflicts, are value conflicts at their root (Hill et al., 2017; IUCN, 2023). Policies and management tactics that effectively address value conflicts need to be grounded in the appropriate social science considerations. Yet, conservation scientists rarely have the social science training needed to develop sociopolitical policy solutions (Frank et al., 2019; IUCN, 2023; Primo & Brooks, 2022). Although this situation is changing, conservation social science is not yet mainstream and wildlife conservation often relies on biological or technical solutions (Bennett, Roth, Klain, Chan, Clark, et al., 2017). Kaplan (1964) referred to this phenomenon as the “law of the instrument,” noting that scientists see problems through the lens of solutions within their skillset. In popular parlance, if one only has a hammer, all problems look like a nail. Bennet, Roth, Klain, Chan, Clark, et al. (2017) identify 4 barriers to mainstreaming the social sciences in conservation—ideological, institutional, knowledge, and capacity. We used a case study of outdoor cat management in Hawaii to illustrate how multiparty efforts grounded in a range of social science disciplines can address these barriers to help break out of policy panacea traps. We illustrate how coproducing science communication, an SES diagnostic framework, and conservation marketing helped parties in conflict identify areas for collaboration, broaden the policy toolbox, and still allow space for policy tools originally framed as opposing panaceas.

## OUTDOOR CAT MANAGEMENT IN HAWAII

Conflicts over outdoor cat management in Hawaii have followed the same general trajectory as conflicts in the rest of the United States but with heightened wildlife conservation concerns for wildlife in Hawaii due to the high level of endemism, increased vulnerability to non‐native predators, and sensitivity to toxoplasmosis (a disease caused by a parasite that only reproduces in felines). A multiparty task force convened in Kauai from August 2013 to February 2014 identified prominent concerns and recommendations (Adler, 2014). However, the group failed to follow through on coordinated implementation, and relationships begun during the process did not last. Since then, we have been involved with conservation agencies and cat welfare organizations that share interests in reducing outdoor cat populations (some were involved in the work on Kauai), but have conflicting beliefs about appropriate methods, time frames, and underlying goals. In previous social science research, we demonstrated how these differences largely stem from each side thinking about outdoor cats as very different types of animals. Wildlife conservation professionals viewed outdoor cats through standards of invasive species management, whereas cat welfare professionals viewed outdoor cats as homeless pets, applying standards for companion animals (Leong et al., 2020). Interviewees for that project were interested in building on this understanding to find ways to work together, which served as a catalyst for our ongoing work.

Over the last 5 years, we collaboratively sought to escape the policy panacea trap focused on humane dispatch versus TNR. A small group meets a few times a year, initially composed of animal welfare professionals from the Hawaiian Humane Society (HHS) on Oahu and wildlife conservation members of the Toxoplasmosis and At‐large Cat Technical Working Group (TACTWG). The TACTWG comprises federal, state, and county organizations in Hawaii with mandates related to addressing the impacts of toxoplasmosis on native wildlife. The HHS‐TACTWG small group set crucial ground rules based on principles of conflict transformation and conservation peacebuilding (Madden & McQuinn, 2017). All had to agree not to eliminate each other's preferred policy options (TNR and humane dispatch) and to focus on agreeable, common ground solutions to reduce impacts from outdoor cats. We codeveloped a set of products informed by the applied social science disciplines of science communication, conflict transformation, and conservation marketing, which has built a strong foundation for coproducing future policy solutions.

## BRIDGING IDEOLOGIES AND KNOWLEDGE WITH THE SCIENCES OF SCIENCE COMMUNICATION

Differing ideologies about how the world works and how people should engage with it can lead to incompatible ways of thinking about a problem and its management (Bennet, Roth, Klain, Chan, Clark, et al., 2017). These incompatibilities can also be based on different knowledge systems, including discipline‐specific training, experience, and methods. We applied the sciences of science communication to help the wildlife conservation and cat welfare professionals understand each other's worldviews and develop a common language. Science communication seeks to convey information about scientific research, discovery, and application in a way that is understandable to nonscientists, and a range of social sciences contributes to effectiveness of communicating science (Fischhoff, 2013; Jamieson et al., 2017).

As a starting point, we developed plain language and analogies to better resonate with how people outside of the conservation world relate to outdoor cats. We also worked with a graphic designer to make our ideas more visually compelling. The HHS‐TACTWG provided feedback on drafts of the graphic, which also helped each side understand the other's nuanced views on the issue. The HHS also helped us reach other members of the animal welfare community for additional feedback, and we leveraged TACTWG connections to present on the topic at conferences and as a case study for a science communication training. As we iteratively modified and improved the graphic, we built trust and developed a shared vocabulary and collaborative working dynamic before discussing specific policy or management activities. This process ensured that all parties understood the broader problem in the same way, saw a place for their solutions, and felt accurately represented.

**FIGURE 1:**
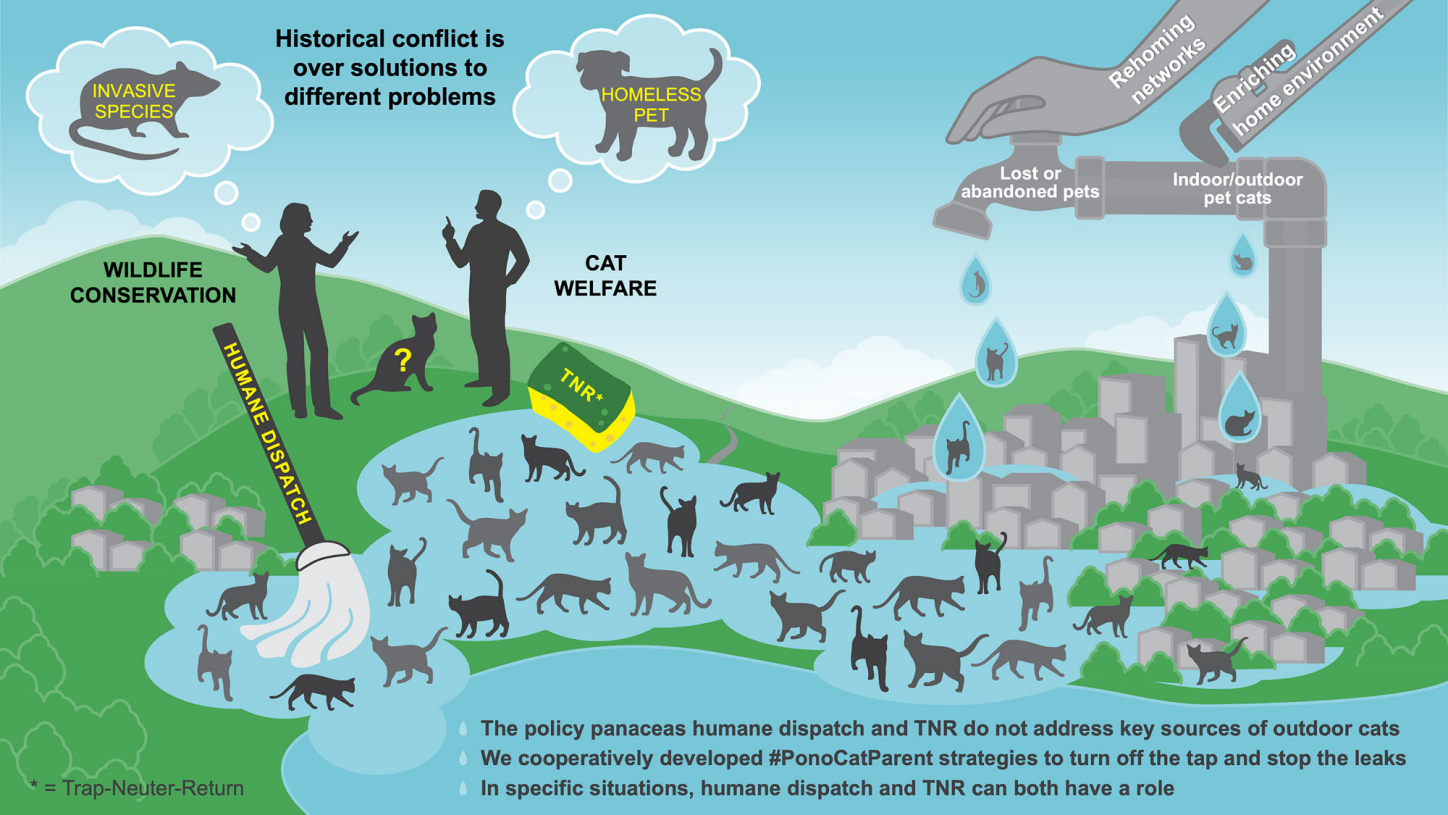
Shared visualization of the full social–ecological system relative to management of outdoor cats.

Figure 1 illustrates the resulting shared visualization of the full SES. It uses the analogy of arguing over whether a mop or sponge is better to control the flood stemming from a leaky faucet to represent the full outdoor cat management system and common policy panaceas. Water in itself is not necessarily damaging, just as cats as a species are not inherently bad. However, long‐standing conflicts focused on only one portion of the system and used different mental models with different goals in mind as the foundation for policy solutions. Wildlife conservation professionals viewed outdoor cats as an invasive species. They advocated for humane dispatch, which refers to methods of killing animals that result in the least amount of suffering and may be applied to healthy animals for population control purposes. It is different from euthanasia, which is used to end the suffering of an animal that cannot be helped with medical care. Euthanasia requires trapping and administering of drugs by a licensed veterinarian. The stress of trapping and administering drugs may be more traumatic (and less humane) than sharpshooting or a bolt to the head in a trap when the intent is to cull the population. Cat welfare professionals, in contrast, viewed many outdoor cats as homeless pets. They promoted euthanasia when animals were clearly suffering and could not be saved, but did not see it as a morally appropriate form of population control. Instead, they advocated for TNR. We used images of a rat and dog to reinforce that the concepts of invasive species and homeless pet are tied to existing cultural referents that are commonly known. We took inspiration from the book *Some We Love, Some We Hate, Some We Eat* (Herzog, 2011), which demonstrates how society establishes unconscious rules, or norms, for how people think about animals. A rat and dog were used as images on the book cover depicting the strongly held societal norms in the United States of rats as hated species and dogs as loved species. This illustrates that when wildlife conservation professionals and cat welfare professionals argue about cats, they are not talking about the same animal.

The mop and sponge illustrate that both humane dispatch and TNR can reduce outdoor cat populations to some degree in certain contexts, such as for functionally closed populations, when attractants are removed and when other options, such as adoptions, are used simultaneously (Wald & Peterson, 2020). Yet, the leaky faucet analogy also makes clear that neither of these policy panaceas can succeed without addressing pet cats that continue to be lost, abandoned, or allowed outside, thereby serving as sources of immigration to existing outdoor cat populations, which has been demonstrated in modeling efforts (Lohr et al., 2012). Reducing the number of pet cats added to the outdoor cat population is a shared goal that wildlife conservation and cat welfare communities agree on and can approach together from a shared set of values. Shifting the key audience to pet owners also broadens the policy space, providing opportunities for wildlife conservation and cat welfare professionals to work together on solutions. Potential solutions (many identified in previous studies) are as follows: require microchips to reunite people with lost pets; provide information to transient people on what to do if they cannot bring their cats with them when they leave the island; and provide guidance on how owners can bond with their cats, create enriching home environments to minimize behavior problems, and reduce abandonment. Entrenched positions pitting humane dispatch and TNR against each other also miss opportunities to identify situations where each method might be appropriate. If all parties can agree on those situations, keeping both management strategies available can begin to reduce the number of outdoor cats rather than induce a stalemate where neither tool is used strategically.

## BUILDING AN SES DIAGNOSTIC FRAMEWORK

As the HHS‐TACTWG group became more comfortable working together, they decided to think more critically about the different categories of cats that have been suggested through social science research conducted by Leong et al. (2020) and Crowley et al. (2019) and codified in other countries, such as New Zealand and Australia (Farnworth et al., 2010). Following standards of practice for conflict transformation (Madden & McQuinn, 2017) and coproduction of knowledge (Norström et al., 2020), we led the group through an iterative process to identify the different characteristics of the outdoor cat SES, detailing the social, environmental, and management contexts that the larger groups collectively addressed. The initial focus was on characterizing all types of cats, following other existing typologies. However, the number and combination of conditions contributing to unique management contexts were too large for a simple typology. Instead, core dimensions of 3 key elements were identified: social, legal, and environmental contexts. Each of these has implications for social and policy constraints and solutions (Table 1). The number of combinations of dimensions and conditions identified in Table 1 illustrates the impossibility of developing a policy panacea that can adequately address every scenario. Instead, Table 1 can be used as a diagnostic tool to help practitioners identify the potential policy implications and considerations relevant to their specific situation. This exercise can also help anticipate potential controversies and identify necessary partners.

**TABLE 1:** Dimensions of outdoor cat management.

Dimension	Common conditions or variables	Implications	Policy considerations
Social context			
Cat ownership	Pet (owned) Semiowned Unowned	Determines who is responsible for the cat	Owned cats are private property. Other cats may be semiowned (e.g., microchip is assigned to a colony caretaker). A good faith effort to determine ownership should be part of any management plan.
Socialization of cats	Socialized (e.g., approaches people, allows touch, meows at people) Less socialized (e.g., might approach, allow touch, or meow) Unsocialized (e.g., does not approach, allow touch, or meow)	Determines if cats will be adoptable	Shelters will generally place socialized cats for adoption but will euthanize or will not accept unsocialized cats. Socialized cats will be even more strongly thought of as homeless pets. If cats that are or can be socialized are present, it is wise to include a plan to adopt them out. Socialization level can be challenging to assess if cats are scared or threatened (e.g., through trapping).
Relationship with human community	Proximity to human settlements Presence of individual or group caretakers Presence of other people concerned about what happens to the cats Presence of other people who view cats as pests or nuisances	Determines what types of caretakers or other interest groups are present and will need to be engaged	People may form associations with cats that live near human settlements. Caretakers will have a vested interest in outcomes for individual cats. Others in the community may have an interest in what happens to cats in a particular area. It will be important to work with any caretakers or community organizations to understand local sentiment toward these cats.
Legal context			
Land ownership responsibility	Cats are on private lands Cats are on public lands Cats are in designated conservation areas (these conditions may be in combination)	Determines who has responsibility for various impacts from cats	Managers, landowners, and caretakers may be liable for impacts from cats. Some areas may have specific mandates that affect outdoor cat management (e.g., restaurants and zoos must consider public health or health of their collections; public lands have policies related to their conservation mission).
Laws or policies about how to manage cats	Any existing restrictions on cat management at various jurisdictional levels	Determines legal authority for management actions toward cats	Municipalities may have defined legal responsibilities around outdoor cats. Any existing laws will need to be followed. If existing laws are not being enforced, consider why not, and what would be required to make them more effective. Consider that a legal approach may not be the most effective one to take.
Laws and policies about how to manage wildlife	Presence of protected species Designated critical habitat for threatened and endangered species	Determines whether there are conservation‐related mandates that need to be considered	Species with special state or federal protected status and areas that are designated as critical habitat have specific requirements that must be met under laws, such as Migratory Bird Treaty Act, Endangered Species Act, Marine Mammal Protection Act, state wildlife action plans, and state wildlife laws.
Environmental context			
Proximity to areas supporting conservation	Presence of sensitive native wildlife or the habitat on which they depend Proximity to protected natural areas	Determines the likelihood of impact to sensitive species or habitats	Sensitive native wildlife species may not be able to withstand cat predation or diseases transmitted by cats. Protected areas or species may be subject to laws and policies outlines above.
Proximity to waterways	Likelihood of feces entering waterways Concentration of cats at locations near water	Determines likelihood of *Toxoplasma* oocysts entering ecosystem	Areas with higher likelihood of runoff and higher concentration of cats are a higher risk to marine mammals, such as Hawaiian monk seals.
Impacts of cat removal on other species	Presence of other invasive species being controlled by cats	Determines whether there might be potential ecosystem cascade effects (especially on islands)	If removal of cats might release pressure on other invasive species (e.g., rats or rabbits), a recommended practice is having a concurrent plan to address those species.

**FIGURE 2:**
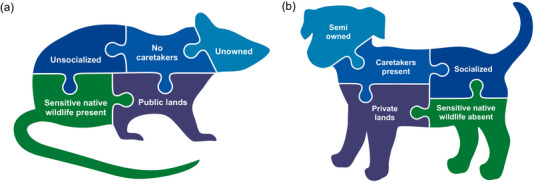
Dimensions of the outdoor cat management context that are the focus of (a) wildlife conservation professionals, who see cats as invasive species, and (b) cat welfare professionals, who see cats as homeless pets.

The development of Table 1 also revealed that wildlife conservation and cat welfare professionals focus on cats in very different management contexts. These management contexts reinforced the views of cats as invasive species or homeless pets (Figure 2). Cat welfare professionals work mostly with cats that have a relationship with human communities, such as stray cats in neighborhoods or cats that are being taken care of in predominantly urban colonies (e.g., at feeding sites). The animal welfare community is less focused on cats born in the wild that have no human contact. However, these unsocialized cats that are not dependent on humans are a particular focus of wildlife conservation professionals because they may have the largest predation impacts on native wildlife (Cove et al., 2018), especially in or near areas of critical habitat for threatened and endangered species, such as seabird colonies (Raine et al., 2020). These 2 situations have very different policy implications, again illustrating the need for solutions matched to the management context.

## CONSERVATION MARKETING CAPACITY BUILDING

With a shared view of the broader overall system, it became easier to identify areas for collaboration. The HHS‐TACTWG small group committed to working together on a campaign targeting pet owners but realized they needed to first build their collective capacity in conservation marketing. Conservation marketing is an emerging discipline that applies social psychology and marketing strategies to develop strategic communication approaches aimed at encouraging proconservation behaviors (Bennett, Roth, Klain, Chan, Christie, et al., 2017; Smith et al., 2020). Conservation practitioners are becoming increasingly aware that information and education alone are rarely adequate to result in desired behaviors and that conservation success relies on behavior change at the societal scale (van Leuvan et al., 2022).

The HHS‐TACTWG small group participated in a conservation marketing training and a series of working sessions to identify desired behaviors and messaging that could help prevent pet cats from becoming outdoor cats. Participating in the training and workshops together gave them practice working collaboratively to include each other's perspectives and built capacity for conservation marketing in general. This insight helped identify the specific owner behaviors that are necessary to prevent indoor pet cats from becoming outdoor cats and develop pilot resources to help pet owners raise their cats in ways that improve the health of cats, wildlife, and Hawaii.

**FIGURE 3:**
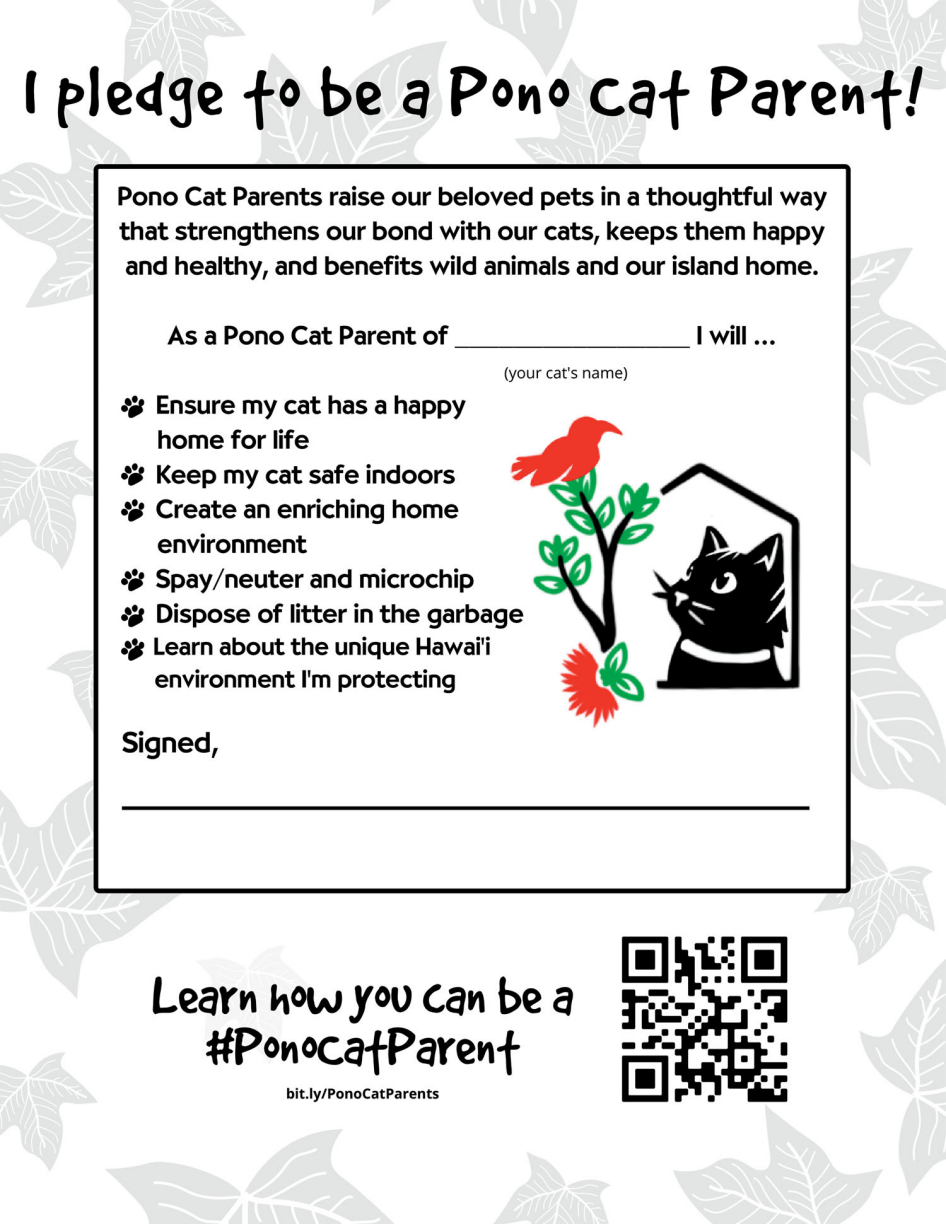
Pono Cat Parent Pledge, which describes core sets of behaviors that can help improve the bond between pet owners and their cats, ensure pet cats are happy and healthy, and protect wildlife and Hawaii.

Six sets of behaviors were identified and organized into a Pono Cat Parents pledge (Figure 3). *Pono* is a Hawaiian word that means appropriate, correct, and deemed necessary by traditional standards in the Hawaiian culture (for full definition, see “pono”, n.d.). It is commonly used in Hawaii and was selected to convey social and cultural norms and expectations appropriate to Hawaii and make the initiative more locally accessible and positive than phrases such as *responsible pet ownership*. The group also consulted with the Pacific Islands Regional Cultural Resources Coordinator for NOAA Office of National Marine Sanctuaries on appropriateness of use and how best to represent the concept throughout the materials (K. Quiocho, Native Hawaiian, lives in Hilo, Hawaii, personal communication, May 31, 2022). The Hawaii Veterinary Medical Association (HVMA) was identified as a neutral authority on pet health, which would be a concern for pet owners and therefore the most appropriate organization to provide the information. Members of the HHS‐TACTWG group already had established relationships with HVMA, which has posted the pledge and associated resources for each set of behaviors on their website (https://hawaiivetmed.org/pono‐pet‐parent/). The HHS‐TACTWG small group has grown to include partners on other islands that jointly support policies for owned pets, such as requiring microchips and providing low‐cost spaying and neutering. They have also identified shared data needs, including research to understand attitudes and behaviors of pet cat owners, a shared methodology for counting cats to determine whether population management strategies are effective, and habitat models to identify areas where higher concentrations of cats or *Toxoplasma* oocysts may have bigger environmental or human health impacts.

## DISCUSSION

Like outdoor cat management, many conservation challenges are value‐based social conflicts at their root. In our case study, efforts grounded in social science helped parties in conflict collaboratively address issues that exacerbate policy panacea traps if not addressed: understanding each other's different ideologies and knowledge systems; diagnostic frameworks that embrace different organizational cultures, interests, histories, and decision‐making structures; and building capacity for behavior change strategies built on a common value system.

In conservation conflicts, attempting to change the value system of others is unlikely to be effective and can have detrimental conservation outcomes (Ford et al., 2021; Manfredo et al., 2017; Wald & Peterson, 2020). Instead, it is important to “embrac[e] the plurality of reality” (Levin et al., 2021, p. 68) to build partnerships around commonly shared values. When we took this approach with outdoor cats, we were able to clearly articulate the full outdoor cat SES and illustrate how TNR and humane dispatch act as polarizing policy panaceas. Switching our focus to pet cats then allowed us to work from a commonly shared set of values toward mutually agreeable, actionable, and localized solutions. It also facilitated relationship and trust building that strengthened the ability to discuss polarizing topics, opening the possibility to addressing other areas of common interest in the future.

This ability to engage in dialogue allowed us to identify where each side was focused on different sets of policy concerns and unaware of each other's core constraints. Articulating the dimensions of outdoor cat management (Table 1) illustrates why a one‐size‐fits‐all policy approach is unlikely to succeed. Instead, society needs diverse policies based on specific situations to break out of policy panacea traps (Cumming, 2018; Levin et al., 2021; Ostrom, 2007). Others have identified the importance of considering multiple social and ecological constraints for outdoor cat management and matching management actions to specific contexts (Kokotovich et al., 2021; Schweitzer & Gillin, 2020; Wald & Peterson, 2020). Different contexts may call for different tactics, and understanding context can identify opportunities for creative cooperation that are crucial to achieving successes (Cumming, 2018).

Shifting our energy toward shared values regarding pet ownership also allowed us to collectively build our capacity for developing behavior change campaigns. Conservation marketing is an emerging discipline that has been gaining momentum since the 2010s. Campaigns targeting cat owners should appeal to concerns about the well‐being of their pet and be delivered by trusted messengers who align with those concerns (van Eeden et al., 2021). Partnerships with the cat welfare community are crucial because they serve as established trusted messengers when wildlife conservation messaging concerns may not resonate with pet cat owners (Linklater et al., 2019; van Eeden et al., 2021). These types of unlikely partnerships become possible when the approach to conflict is shifted to a focus on positive interactions between wildlife and humans, as illustrated in the case studies compiled in Frank et al. (2019).

The group in Hawaii identified a number of key lessons that were crucial to developing productive partnerships. First, it was important to establish that neither group would work to eliminate the other's core strategy (TNR or humane dispatch). This allowed the time to work up to more difficult conversations. Second, committing to working together on areas of shared interest (affecting how people view responsible cat ownership) opened the possibility for broader dialogues on a larger range of strategies. This led to the development of Table 1 and discussions about future collaboration. Third, approaching interactions with the intent to learn from each other rather than trying to convert each other built trust, created opportunity, and provided occasions to actively listen and engage without fear of confrontation. Although much future work still remains to develop the full set of resources for the Pono Cat Parent campaign and to conduct research and craft legislation that meets shared needs and specific contexts, progress is now possible and the group is beginning to have those more difficult discussions in a productive way.

These lessons represent core tenets of conservation conflict transformation (Madden & McQuinn, 2017) applied to human–wildlife conflict. In recent years, a body of literature has emerged that consolidates these and other learnings into principles for addressing human–wildlife conflicts and guidance on how to put them into practice (Frank et al., 2019; Hill et al., 2017; IUCN, 2023). These resources are also related to the establishment of the IUCN SSC Human–Wildlife Conflict & Coexistence Specialist Group in 2016 and the section “Human–Wildlife Interactions” in the journal *Frontiers in Conservation Science*, established in 2020. Coexistence requires self‐awareness from conservationists (IUCN, 2023). In our experience, a crucial first step was self‐awareness that we were mired in a policy panacea trap. Once there is recognition of a need for a broader set of tools, this growing body of resources can be more readily accessed and applied.

The groups in Hawaii that were involved in the processes described above were not the ones who introduced the bills in 2022 and had little control over public debates that predictably followed established arguments for or against humane dispatch and TNR. The bill, HB1987, which proposed counting cats in order to cull them, received over 1300 pages of largely negative public comment and was quickly deferred indefinitely. Initially, SB2837 was progressing well through the legislative process. It was relatively neutral, funding low‐cost spaying and neutering for any situation, including TNR. However, after HB1987 failed to move forward, SB2837 was amended to require that funds be used primarily for TNR and to add counting cats. After 2 amendments in the House and 2 in the Senate, the bill never made it out of conference. Both counting cats and low‐cost spaying and neutering are management actions that would help all parties. However, once they became attached to established policy panaceas based on conflicting sets of values, neither was supported. By the time the members of the HHS‐TACTWG learned of these efforts, it was too late to change the trajectory of the policy panacea arguments on either side, which prevented productive edits to the legislation and resulted in inaction.

In July 2023, a request was brought forward to the Hawaii Board of Agriculture to change the listing of the hybrid Savannah cat crosses of the serval (*Felis leptailurus*) with the domestic cat from the list of prohibited animals to enable individual possession. The HHS brought this agenda item to the attention of the TACTWG, and all organizations worked on opposing testimony, some on the grounds of animal welfare, and some on the grounds of conservation. The petitioner's request to allow hybrid cats to be imported was denied. This experience demonstrated that the groups can work together, and they are now collaborating to craft language for policies that all parties can support. Further, they recognized a core need to help policy makers avoid naively activating policy panacea traps and craft language that can be universally supported. These types of policy solutions are not only less likely to incite conflict but also more likely to gain political support, as are any bipartisan agreements.

Based on our experiences, we suggest that others who find themselves enmeshed in value‐based conservation conflicts utilize social science insights to transform how conflict is addressed by embracing each other's conflicting realities and values rather than trying to change them. Identifying shared values and broadening perspectives to acknowledge the full SES can help groups in conflict move beyond policy panaceas that end in stalemate to develop shared understandings of context‐specific policies and identify opportunities for creative cooperation that make real conservation progress.

## References

[cobi14321-bib-0001] Adler, P. (2014). Feral cat task force: Findings & recommendations . Accord 3.0. https://web.archive.org/web/20170403093550/http://www.accord3.com/docs/FCTF%20Report%20FINAL.pdf

[cobi14321-bib-0002] Balint, P. J. , Stewart, R. E. , Desai, A. , & Walters, L. C. (2011). Wicked environmental problems: Managing uncertainty and conflict. Island Press.

[cobi14321-bib-0003] Bennett, N. J. , Roth, R. , Klain, S. C. , Chan, K. M. , Clark, D. A. , Cullman, G. , Epstein, G. , Nelson, M. P. , Stedman, R. , Teel, T. L. , Thomas, R. E. , Wyborn, C. , Curran, D. , Greenberg, A. , Sandlos, J. , & Veríssimo, D. (2017). Mainstreaming the social sciences in conservation. Conservation Biology, 31(1), 56–66. 27334309 10.1111/cobi.12788

[cobi14321-bib-0004] Bennett, N. J. , Roth, R. , Klain, S. C. , Chan, K. , Christie, P. , Clark, D. A. , Cullman, G. , Curran, D. , Durbin, T. J. , Epstein, G. , Greenberg, A. , Nelson, M. P. , Sandlos, J. , Stedman, R. , Teel, T. L. , Thomas, R. , Veríssimo, D. , & Wyborn, C. (2017). Conservation social science: Understanding and integrating human dimensions to improve conservation. Biological Conservation, 205, 93–108.

[cobi14321-bib-0005] Brock, W. A. , & Carpenter, S. R. (2007). Panaceas and diversification of environmental policy. Proceedings of the National Academy of Sciences of the United States of America, 104(39), 15206–15211. 17881581 10.1073/pnas.0702096104 PMC2000546

[cobi14321-bib-0006] Cove, M. V. , Gardner, B. , Simons, T. R. , Kays, R. , & O'Connell, M. F. (2018). Free‐ranging domestic cats (*Felis catus*) on public lands: Estimating density, activity, and diet in the Florida Keys. Biological Invasions, 20, 333–344.

[cobi14321-bib-0007] Crowley, S. L. , Cecchetti, M. , & McDonald, R. A. (2019). Hunting behaviour in domestic cats: An exploratory study of risk and responsibility among cat owners. People and Nature, 1, 18–30.

[cobi14321-bib-0008] Cumming, G. (2018). A review of social dilemmas and social‐ecological traps in conservation and natural resource management. Conservation Letters, 11(1), Article e12376.

[cobi14321-bib-0009] Farnworth, M. J. , Dye, N. G. , & Keown, N. (2010). The legal status of cats in New Zealand: A perspective on the welfare of companion, stray, and feral domestic cats (*Felis catus*). Journal of Applied Animal Welfare Science, 1, 180–188. 10.1080/10888700903584846 20349383

[cobi14321-bib-0010] Fischhoff, B. (2013). The sciences of science communication. Proceedings of the National Academy of Sciences of the United States of America, 110(suppl.3), 14033–14039. 23942125 10.1073/pnas.1213273110 PMC3752164

[cobi14321-bib-0011] Ford, A. T. , Ali, A. H. , Colla, S. R. , Cooke, S. J. , Lamb, C. T. , Pittman, J. , Shiffman, D. S. , & Singh, N. J. (2021). Understanding and avoiding misplaced efforts in conservation. Facets, 6, 252–271. 10.1139/facets-2020-0058

[cobi14321-bib-0012] Frank, B. , Glikman, J. A. , & Marchini, S. (Eds.). (2019). Human‐wildlife interactions: Turning conflict into coexistence. Cambridge University Press.

[cobi14321-bib-0013] Herzog, H. (2011). Some we love, some we hate, some we eat. HarperCollins Publishers.

[cobi14321-bib-0014] Hill, C. M. , Webber, A. D. , & Priston, N. E. C. (Eds.). (2017). Understanding conflicts about wildlife: A biosocial approach. Berghahn Books.

[cobi14321-bib-0015] International Union for Conservation of Nature (IUCN) . (2023). IUCN SSC guidelines on human‐wildlife conflict and coexistence (1st ed.). https://portals.iucn.org/library/node/50756

[cobi14321-bib-0016] Jamieson, K. H. , Kahan, D. M. , & Scheufele, D. (Eds.). (2017). The Oxford handbook of the science of science communication. Oxford University Press.

[cobi14321-bib-0017] Kaplan, A. (1964). The conduct of inquiry: Methodology for behavioral science. Chandler Publishing Company.

[cobi14321-bib-0018] Kokotovich, A. E. , Delborne, J. A. , Redford, K. , Cook, T. , Leslie, E. , Sieracki, J. , & Trevino, D. (2021). Free‐ranging cats: Understanding conflict and the potential for engagement (Natural Resource Report NPS/NRSS/BRD/NRR—2021/2297). National Park Service. 10.36967/nrr-2287250

[cobi14321-bib-0019] Leong, K. M. , Gramza, A. R. , & Lepczyk, C. A. (2020). Understanding conflicting cultural models of outdoor cats to overcome conservation impasse. Conservation Biology, 34(5), 1190–1199. 32374059 10.1111/cobi.13530 PMC7540411

[cobi14321-bib-0020] Levin, P. S. , Gray, S. A. , Möllman, C. , & Steir, A. C. (2021). Perception and conflict in conservation: The Roshemon effect. Bioscience, 71(1), 64–72.

[cobi14321-bib-0021] Linklater, W. L. , Farnworth, M. J. , van Heezik, Y. , Stafford, K. J. , & MacDonald, E. A. (2019). Prioritizing cat‐owner behaviors for a campaign to reduce wildlife depredation. Conservation Science and Practice, 1(5), Article e29.

[cobi14321-bib-0022] Lohr, C. A. , Cox, L. J. , & Lepczyk, C. A. (2012). Costs and benefits of trap‐neuter‐release and euthanasia for removal of urban cats in Oahu, Hawaii. Conservation Biology, 27(1), 64–73. 23009077 10.1111/j.1523-1739.2012.01935.x

[cobi14321-bib-0023] Loss, S. R. , & Marra, P. P. (2018). Merchants of doubt in the free‐ranging cat conflict. Conservation Biology, 32(2), 265–266. 29377342 10.1111/cobi.13085

[cobi14321-bib-0024] Lynn, W. S. , Santiago‐Ávila, F. , Lindenmayer, J. , Hadidian, J. , Wallach, A. , & King, B. J. (2019). A moral panic over cats. Conservation Biology, 33(4), 769–776. 31087701 10.1111/cobi.13346 PMC6852131

[cobi14321-bib-0025] Madden, F. , & McQuinn, B. (2017). Conservation conflict transformation: Addressing the missing link in wildlife conservation. In C. M. Hill , A. D. Webber , & N. E. C. Priston (Eds.), Understanding conflicts about wildlife: A biosocial approach (pp. 148–169). Berghahn Books.

[cobi14321-bib-0026] Manfredo, M. J. , Bruskotter, J. T. , Teel, T. L. , Fulton, D. , Schwartz, S. H. , Arlinghaus, R. , Oishi, S. , Uskul, A. K. , Redford, K. , Kitayama, S. , & Sullivan, L. (2017). Why social values cannot be changed for the sake of conservation. Conservation Biology, 31(4), 772–780. 27757996 10.1111/cobi.12855

[cobi14321-bib-0027] Marra, P. P. , & Santella, C. (2016). Cat wars: The devastating consequences of a cuddly killer. Princeton University Press.

[cobi14321-bib-0028] Mascia, M. B. , Brosius, J. P. , Dobson, T. A. , Forbes, B. C. , Horowitz, L. , McKean, M. A. , & Turner, L. A. (2003). Conservation and the social sciences. Conservation Biology, 17, 649–650.

[cobi14321-bib-0029] Nie, M. A. (2003). Beyond wolves: The politics of wolf recovery and management (NED‐New ed.). University of Minnesota Press.

[cobi14321-bib-0030] Norström, A. V. , Cvitanovic, C. , Löf, M. F. , West, S. , Wyborn, C. , Balvanera, P. , Bednarek, A. T. , Bennett, E. M. , Biggs, R. , de Bremond, A. , Campbell, B. M. , Canadell, J. G. , Carpenter, S. R. , Folke, C. , Fulton, E. A. , Gaffney, O. , Gelcich, S. , Jouffray, J.‐B. , Leach, M. , … Österblom, H. (2020). Principles for knowledge co‐production in sustainability research. Nature Sustainability, 3, 182–190. 10.1038/s41893-019-0448-2

[cobi14321-bib-0031] Noss, R. (2007). Values are a good thing in conservation biology. Conservation Biology, 21(1), 18–20. 17298505 10.1111/j.1523-1739.2006.00637.x

[cobi14321-bib-0032] Ostrom, E. (2007). A diagnostic approach for going beyond panaceas. Proceedings of the National Academy of Sciences of the United States of America, 104(39), 15181–15187. 17881578 10.1073/pnas.0702288104 PMC2000497

[cobi14321-bib-0033] Ostrom, E. , Janssen, M. A. , & Anderies, J. M. (2007). Going beyond panaceas. Proceedings of the National Academy of Sciences of the United States of America, 104(39), 15176–15178. 17881583 10.1073/pnas.0701886104 PMC2000490

[cobi14321-bib-0034] Primo, J. V. , & Brooks, J. J. (2022). Is that a thing? Applied social scientists in federal resources management. Practicing Anthropology, 44(2), 16–20.

[cobi14321-bib-0035] Raine, A. F. , Driskill, S. , Vynne, M. , Harvey, D. , & Pias, K. (2020). Managing the effects of introduced predators on Hawaiian endangered seabirds. Journal of Wildlife Management, 84, 425–435.

[cobi14321-bib-0036] Rittel, H. W. J. , & Webber, M. M. (1973). Dilemmas in a general theory of planning. Policy Sciences, 4, 155–169.

[cobi14321-bib-0037] Schweitzer, S. H. , & Gillin, C. M. (Eds.). (2020). Toolkit to address free‐ranging domestic cats (Felis catus) on agency lands managed for native wildlife and ecosystem health. AFWA Feral and Free‐ranging Cat Working Group. https://www.fishwildlife.org/application/files/7216/1919/5140/Cat‐Toolkit‐v7‐Print.pdf

[cobi14321-bib-0038] Smith, R. , Salazar, G. , Starinchak, J. , Thomas‐Walters, L. , & Veríssimo, D. (2020). Social marketing and conservation. In W. Sutherland , P. Brotherton , Z. Davies , N. Ockendon , N. Pettorelli , & J. Vickery (Eds.), Conservation research, policy and practice (Ecological reviews) (pp. 309–322). Cambridge University Press. 10.1017/9781108638210.019

[cobi14321-bib-0039] van Eeden, L. M. , Hames, F. , Faulkner, R. , Geschke, A. , Squires, Z. E. , & McLeod, E. M. (2021). Putting the cat before the wildlife: Exploring cat owners' beliefs about cat containment as predictors of owner behavior. Conservation Science and Practice, 3(10), Article e502.

[cobi14321-bib-0040] van Leuvan, N. , Highleyman, L. , Fujita, R. , & Kellerman, A. (2022). Making shift happen: Designing for successful environmental behavior change. New Society Publishers.

[cobi14321-bib-0041] Wald, D. M. , & Peterson, A. L. (2020). Cats and conservationists: The debate over who owns the outdoors. Purdue University Press.

